# Butremycin, the 3-Hydroxyl Derivative of Ikarugamycin and a Protonated Aromatic Tautomer of 5′-Methylthioinosine from a Ghanaian *Micromonospora* sp. K310

**DOI:** 10.3390/md12020999

**Published:** 2014-02-14

**Authors:** Kwaku Kyeremeh, Kojo Sekyi Acquah, Anil Sazak, Wael Houssen, Jioji Tabudravu, Hai Deng, Marcel Jaspars

**Affiliations:** 1 Marine and Plant Laboratory of Ghana, Department of Chemistry, University of Ghana, Accra, P.O. Box LG 56, Ghana; E-Mail: ksaquah@yahoo.com; 2 Department of Biology, Faculty of Art and Science, Ondokuz Mayis University, Kurupelit Samsun 55139, Turkey; E-Mail: asazak@omu.edu.tr; 3 Marine Biodiscovery Centre, Department of Chemistry, University of Aberdeen, Old Aberdeen AB24 3UE, Scotland, UK; E-Mails: w.houssen@abdn.ac.uk (W.H.); j.tabudravu@abdn.ac.uk (J.T.); h.deng@abdn.ac.uk (H.D.); 4 Institute of Medical Sciences, University of Aberdeen, Aberdeen AB25 2ZD, Scotland, UK

**Keywords:** *Micromonospora*, macrolactam, tautomer, tetramic acid, mangroves

## Abstract

A new actinomycete strain *Micromonospora* sp. K310 was isolated from Ghanaian mangrove river sediment. Spectroscopy-guided fractionation led to the isolation of two new compounds from the fermentation culture. One of the compounds is butremycin (**2**) which is the (3-hydroxyl) derivative of the known Streptomyces metabolite ikarugamycin (**1**) and the other compound is a protonated aromatic tautomer of 5′-methylthioinosine (MTI) (**3**). Both new compounds were characterized by 1D, 2D NMR and MS data. Butremycin (**2**) displayed weak antibacterial activity against Gram-positive *S. aureus* ATCC 25923, the Gram-negative *E. coli* ATCC 25922 and a panel of clinical isolates of methicillin-resistant *S. aureus* (MRSA) strains while **3** did not show any antibacterial activity against these microbes.

## 1. Introduction

Mangroves are unique woody plant communities that occur as a convergence place for both marine and terrestrial organisms around the intertidal coasts of tropical and sub-tropical regions. These habitats have been found to be a great assembly point of novel actinomycetes belonging to several genera including *Actinomadura*, *Microbispora*, *Nonomuraea*, *Actinoplanes*, *Micromonospora*, *Verrucosispora*, *Arthrobacter*, *Isoptericola*, *Micrococcus*, *Microbacterium*, *Nocardia*, *Rhodococcus*, *Streptomyces* and many more [1]. Novel metabolites with anti-infective, antitumour, anti-diabetic activity and anti-neurodegenerative activity have been isolated from these genera of actinomycetes obtained from different marine habitats. For example, *Actinomadura* sp. produce the anticancer agent IB-00208 and the chandrananimycins which possess antibacterial, anticancer and antifungal activity [2,3]. *Verrucocispora* sp. is noted for the abyssomicins which have antibacterial activity [4]. Caprolactones and chinikomycins are potent anticancer agents that have been isolated from *Streptomyces* sp. [5,6]. From *Micromonospora* sp. comes the diazepinomicin (ECO-4601) which is known to have antibacterial, anticancer and anti-inflammatory activities [7]. These examples are by no means a comprehensive list of all promising lead molecules isolated from actinomycetes, but underline that continued research of un- or underexplored mangrove ecosystems should yield many novel species of actinomycetes which as a result of their high genomic and metabolic diversity are most likely to produce new drug prototypes. 

Similar ideas have driven the research in our laboratory to investigate mangroves in the Western Region of Ghana for possible isolation of new or novel marine actinomycetes. Several new species of *Actinomyces*, *Streptomyces*, *Salinospora*, *Verrucocispora* and *Micromonospora* have been isolated from the Western Region mangroves using casein starch with sea salts as culture media. One of these species *Micromonospora* sp. K310, was found to produce under the current growth media and conditions the [3-hydroxyl] derivative of ikarugamycin, named butremycin in recognition of the river from which the source organism was obtained and a protonated aromatic tautomer of 5′-methylthioinosine (**3**) complexed to *sec*-butoxide as a counter ion. 

**Figure 1:**
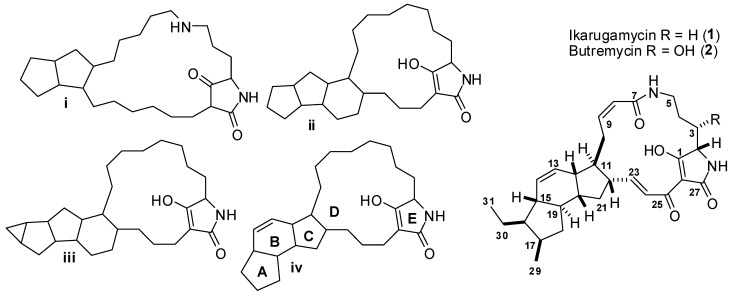
Characteristic ring sequences for polycyclic tetramic acid macrolactams (PTM) compounds isolated to date including ikarugamycin and butremycin.

Butremycin belongs to the family of polycyclic tetramic acid macrolactams (PTM) that possess typical characteristic ring sequences as shown for the structure skeleton i–iv in Figure 1, where the last ring is normally the five membered ring of tetramic acid. Butremycin belongs to the category of compounds that possess the structure skeleton iv and in addition it has a 3-hydroxyl group which has been previously found in all PTMs reported so far with the exception of ikarugamycin [8,9]. Butremycin is the hydroxyl derivative of ikarugamycin and 3-hydroxylation is a feature common to some polycyclic tetramic acid macrolactams found in phylogenetically diverse organisms. The effect of 3-hydroxylation on the bioactivity of these tetramic acid macrolactams has been demonstrated by the work of Li *et al*. [8] but, to the best of our knowledge this is the first report of a PTM from a *Micromonospora* sp. 

## 2. Results and Discussion

### 2.1. Sediment Sample Collection Sites

The Western Region of Ghana is particularly noted for large stretches of mangroves that have become characteristically embedded in the life of the natives. An important river, called the Butre, cuts across a large section of these mangroves before entering the sea. We collected sediment samples from the river at four different sites approximately 100m apart. *Micromonospora* sp. K310 was isolated from one of these sediments (coordinates: 4°49′43.73″N and 1°54′50.84″W). 

### 2.2. Taxonomy of Strain K310 (Genbank Number KF803252)

**Figure 2:**
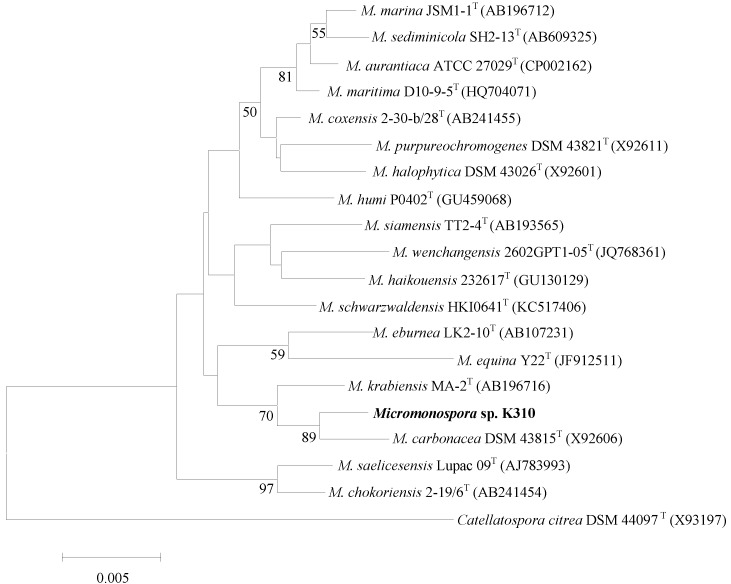
Neighbor-joining tree [10] based on almost complete 16 rDNA gene sequences (1455 nt) showing the position of *Micromonospora* sp. K310 (KF803252) amongst its phylogenetic neighbors. *Catellatospora citrea* DSM 44097^T^ (GenBank accession no. X93197) was used as an out-group. Numbers at nodes indicate the levels of bootstrap support (%); only values >50% are shown. GenBank accession numbers are given in parentheses. Bar, 0.005 substitutions per site.

The organism exhibited a range of chemotaxonomic and phenotypic properties typical of members of the genus *Micromonospora*. An almost complete 16S rDNA gene sequence (1455 nt) was determined for the organism. Primary sequence analysis with the sequences of representatives of the family *Micromonosporaceae* confirmed that the unknown isolate was closely related to the species of the genus *Micromonospora*. The phylogenetic tree based on the neighbor-joining algorithm showed that the strain K310 formed a cluster with *M. carbonacea* DSM 43815^T^ and *M. krabiensis* MA-2^T^ among members of the genus *Micromonospora* (Figure 2). Strain K310 shared 16S rDNA gene sequence similarities of 99.45% (8 nt differences at 1449 locations), 99.03% (14 nt differences at 1436 locations) with *M. carbonacea* DSM and *M. krabiensis* MA-2^T^ respectively. Sequence similarities with all other members of the genus *Micromonospora* were <98.90%. 

### 2.3. Structure Determination of Compound **2** (Butremycin)

A seven-day grown seed culture of *Micromonospora* sp. K310 was used in the inoculation of two L starch casein media in the presence of the Diaion HP-20 resin (50 g/L medium). After 28-day fermentation, the aqueous solution was filtered and HP20 resin was extracted with methanol (3 × 500 mL). The crude extract was concentrated under vacuum, followed by partition using a modified Kupchan method [11]. The purification was achieved through Sephadex LH-20 chromatography followed by semipreparative reversed-phase HPLC resulting in the isolation of **2** (46.6 mg) and **3** (57.0 mg).

Butremycin was isolated as pale yellow crystals soluble in CH_3_OH. The HRESIMS of this compound gave *m*/*z* = 495.2781 [M + H]^+^ corresponding to a molecular formula of C_29_H_39_N_2_O_5_ (Δ = −0.5 ppm) and 12 degrees of unsaturation. Molecular formulae for all fragments emanating from the molecular ion were obtained and used to confirm the subsequent structure obtained from NMR (Supplementary Figure S1). 

**Figure 3:**
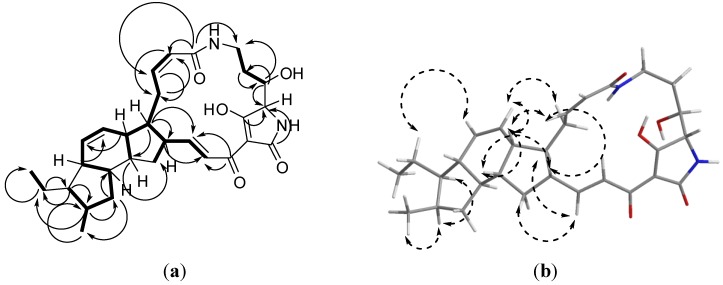
(**a**) Selected COSY (**▬**) and gHMBCAD (**→**) data for butremycin; (**b**) Selected ROESY (↔) data for butremycin (**2**).

Analysis of the ^1^H, ^13^C and multiplicity edited gHSQCAD spectra suggested the presence of 5 quaternary, 16 methine, 6 methylene and 2 methyl carbons. Detailed analysis of the gCOSY spectrum provided substructures as illustrated in Figure 3a. The presence of six olefinic protons at δ_H_ 7.30 (1H, d, *J* = 15.2 Hz, H-24), 6.47 (1H, dd, *J* = 10.0, 15.1 Hz, H-23), 6.02 (1H, ddd, *J* = 2.6, 11.4, 11.4 Hz, H-9), 5.83 (1H, ddd, *J* = 2.1, 2.1, 10.0 Hz, H-14), 5.76 (1H, dd, *J* = 2.8, 11.4 Hz, H-8), 5.62 (1H, ddd, *J* = 2.8, 2.8, 9.9 Hz, H-13) was direct indication of the presence of three double bonds: H-8/H-9 (*cis*), H-13/H-14 (*cis*) and H-23/H-24 (*trans*). Subsequently, using these isolated olefins as starting points, sub-structure extensions were achieved by looking at gHMBCAD peaks from C-23 to H-22 and H-21 with confirmations from the gCOSY cross-peaks between H-21/H-22 and H-22/H-23. Similarly, gCOSY cross-peaks between H-11/H-12, H-12/H-13, H-13/H-14 and H-14/H-15 provided an extension of the C-13/C-14 olefin sub-structure. Also, sub-structure extensions for the C-8/C-9 olefin bond were obtained by inspecting the gCOSY correlations H-8/H-10, H-9/H-10, H-10/H-11 with confirmation from gHMBCAD correlations C-8 to H-10, C-9 to H-10, C-10 to H-8, and C-11 to both H-9 and H-10. From these substructures it became easier to follow the comprehensive spin system present in butremycin with the A5:B6:C5 ring structures (**3a**) obtained by 2D correlations H-11/H-22, C-11/H-22, H-12/H-20, H-15/H-19 and C-15/H-19. 

The tetramic acid structure was constructed by analyzing the gHMBCAD correlations from C-1 (δ_C_ 195.5) to H-2, C-27 (δ_C_ 179.9) to H-2 and C-3 (δ_C_ 72.5) to H-2 and comparison to reference shifts for comparable tetramic acids [12]. All the NMR data for this compound are summarized in Table 1 and raw data can be obtained from Supplementary Figures S2–S7. 

The majority of the stereochemistry is identical to that reported for ikarugamycin, and the ROESY correlations observed confirm this proposal (Figure 3). The only remaining issue is the stereochemistry at C-3. The small coupling constant between H-2 and H-3 (*ca.* 2 Hz) is indicative of a nearly orthogonal relationship between C-2–H-2 and C-3–H-3. This is consistent with a local 2S*, 3S* stereochemistry. The alternative, a 2S*, 3R* gives rise to an anti-periplanar arrangement of C-2–H-2 and C-3–H-3 which should result in a large coupling constant between H-2 and H-3. This suggests that the overall relative stereochemistry for butremycin is 2S*, 3S*, 11S*, 12R*, 15S*, 16R*, 17R*, 19R*, 20R*, 22R*.

**Table 1:** ^1^H and ^13^CNMR data of butremycin in CD_3_OD. δ in ppm, *J* in Hz.

Position	δ_H_ mult (*J* Hz)	δ_C_ mult	HMBC
1		195.5, C	2
2	3.70, d (2.4)	68.4, CH	
3	3.86, ddd (7.2, 1.9, 1.9)	72.5, CH	2, 4, 5
4	1.51, m; 1.24, m	32.1, CH_2_	2, 3
5	3.42, m; 2.71, t (12.0)	37.9, CH_2_	3
6-NH	8.43, s		
7		168.9, C	5, 8, 9
8	5.76, dd (11.4, 2.8)	124.8, CH	10
9	6.02, ddd (11.4, 11.4, 2.6)	141.8, CH	10
10	3.53, m; 2.09, m	25.9, CH_2_	8
11	1.35, m	50.3, CH	9, 10, 20, 22, 23
12	2.51, m	43.4, CH	10, 14
13	5.62, ddd (9.9, 2.8, 2.8)	129.6, CH	
14	5.83, dt (10.0, 2.1)	131.9, CH	
15	1.48, m	48.4, CH	13, 14, 19
16	1.29, m	48.5, CH	17, 30
17	2.20, m	34.3, CH	18, 29, 30
18	2.05, m; 0.63, ddd (11.9, 11.8, 6.8)	39.9, CH_2_	19, 29
19	1.08, m	50.4, CH	14, 18, 21
20	2.00, m	43.2, CH	13, 18, 21
21	2.01, m; 1.10, m	38.1, CH_2_	19, 22, 23
22	2.32, m	49.6, CH	21, 23, 24
23	6.47, dd (15.1, 10.0)	147.8, CH	21, 22
24	7.30, d (15.2)	130.4, CH	22
25		184.7, C	23, 24
26		102.8, C	
27		179.9, C	2
28-NH	8.43, s		
29	0.81, d (6.0)	18.1, CH_3_	17, 18
30	1.42, m; 1.30, m	22.7, CH_2_	16, 31
31	0.87, t (7.1)	13.7, CH_3_	30

### 2.4. Structure Determination of Compound **3**

**Figure 4:**
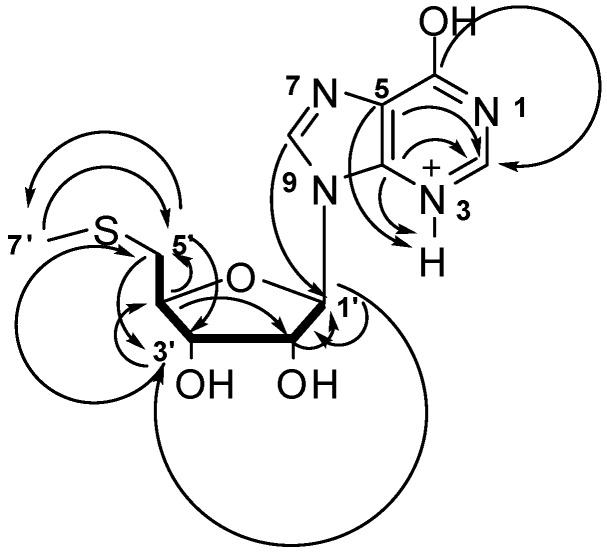
Selected COSY (**▬**) and gHMBCAD (**→**) data for compound **3**.

Compound **3** was isolated as a 1:1 complex with *sec*-butoxide which is most likely to come from the 2-butanol used in the solvent partition process. This compound **3**/*sec*-butoxide complex was yellow and formed crystalline material soluble in CH_3_OH. The HRESIMS of this compound gave *m*/*z* = 299.0810 [M]^+^ corresponding to a molecular formula of C_11_H_15_N_4_O_4_S^+^ and 7 degrees of unsaturation. Analysis of the ^1^H, ^13^C and multiplicity edited gHSQCAD spectrum confirmed the presence of 3 quaternary, 6 methine, 1 methylene and 1 methyl carbons. The δ_C_ 90.23, 75.22, 73.98, 85.65 and 37.47 ppm corresponding to C-1′, C-2′, C-3′, C-4′ and C-5′ respectively, were very characteristic of a series of multiple oxygenations commonly found in sugars. Analysis of the ^1^H and ^13^C NMR provided proof of the presence of a five-membered ring sugar moiety, which could possibly be a furanose. Detailed analysis of the gCOSY spectrum provided the spin system encompassing H-1′ to H-5′ which was extended to include Me-7′ via the the gHMBC correlation from C-5′ to H-7′ (Figure 4). The characteristic ^13^C NMR chemical shifts δ_C_ C-6 (158.9), C-4 (150.2), C-5 (125.8) and C-8 (140.9) taken together with the gHMBCAD correlations C-6 to H-2, C-5 to H-2 and NH-3, C-4 to H-2, NH-3 suggested a protonated adenine ring. The ribose unit and the adenine ring were connected using two key gHMBCAD correlations, C-4 to H-1′ and C-8 to H1′. All NMR data obtained for this compound are summarized in Table 2 and raw data provided in Supplementary Figures S8–S12.

**Table 2:** ^1^H and ^13^C NMR data of compound **3** in CD_3_OD. δ in ppm, *J* in Hz.

Position	δ_H_ mult (*J* Hz)	δ_C_ mult	HMBC
1-N			
2	8.07, s	146.8, CH	
3-NH	8.29, s		
4		150.2, C	2, NH-3,1′
5		125.8, C	2, NH-3
6		158.9, C	2
7-N			
8		140.9, CH	1′
9-N			
1′	6.02, d (4.8)	90.2, CH	2′,3′
2′	4.73, t (5.1)	75.2, CH	1′
3′	4.32, t (4.8)	73.9, CH	4′,5′
4′	4.22, q (5.4)	85.6, CH	2′,5′
5′	2.93, dd (14.1, 5.5) 2.873, dd (14.1, 5.9)	37.5, CH_2_	3′,7′
6′-S			
7′	2.14, s	16.5, CH_3_	5′

### 2.5. Possible Biosynthesis of Compound **3**

**Scheme 1:**
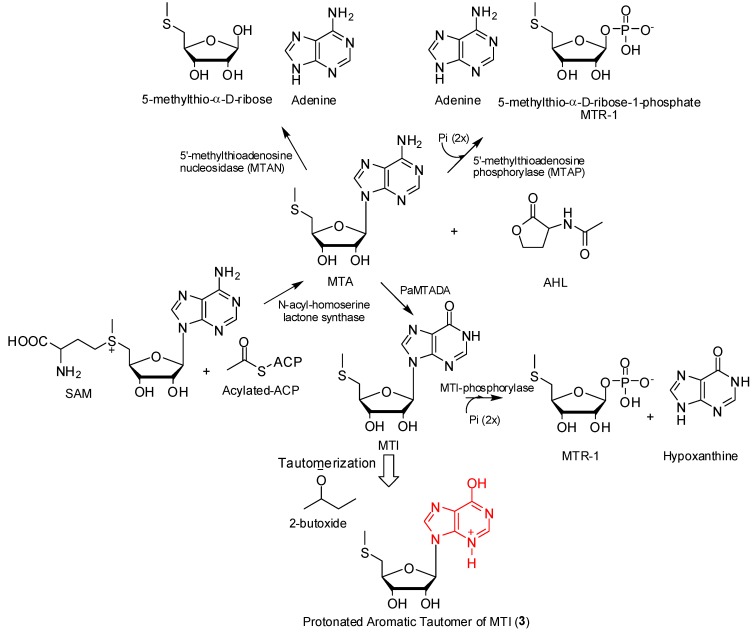
Possible biosynthetic origin of compound (**3**) showing 5′-methylthioadenosine (MTA) degradation pathways in Phyla Bacteria, Eucarya and Archaea and *Pseudomonas aeruginosa*.

Organisms from the Phyla Bacteria, Eucarya and Archaea all require small molecular entities or signal molecules like *N*-acyl-homoserine lactone (AHL) for intercellular communication or quorum sensing [13]. In order to facilitate the perpetuation of complex community behaviors like the secretion of proteins, motility, virulence factor production, and plasmid transfer or biofilm maturation, these organisms rely mainly on quorum sensing [14]. AHL is biosynthesized from *S*-adenosyl methionine (SAM) and acylated-acyl carrier protein (A-ACP) in the presence of LuxI enzymes or *N*-acyl-homoserine lactone synthase with the production of 5′-methylthioadenosine (MTA) as by-product (Scheme 1) [13]. MTA negatively regulates the reactions leading to the formation of AHL through inhibition of AHL synthase [13]. Hence the efficient removal of MTA is essential in any sort of attempt to create a build-up of AHL. Typical one-step MTA degradation pathways are shown in Scheme 1 for Bacteria, Eucarya and Archaea [15]. Recently, Guan *et al.* [15] reported an unusual two-step MTA degradation pathway for *Pseudomonas aeruginosa* (Scheme 1) that involves the deamination aided by a 5′-methylthioadenosine deaminase (PaMTADA) to 5′-methylthioinosine (MTI) followed by *N*-ribosyl phosphorolysis to hypoxanthine and 5-methylthio-α-d-ribose 1-phosphate (MTR-1) [15]. In the course of the current study, we have isolated and characterized a protonated aromatic tautomer of MTI (compound **3**) from *Micromonospora* sp. K310 in considerable quantities. It is possible that this molecule may be an indicator of the presence of this unique two-step MTA degradation pathway previously reported for *Pseudomonas aeruginosa* in *Micromonospora* sp. K310 and hence further studies are being conducted. 

### 2.6. Antibacterial Activity of Butremycin

Butremycin was tested for antibacterial activity against the Gram-positive *S. aureus* ATCC 25923, the Gram-negative *E. coli* ATCC 25922 and a panel of clinical isolates of methicillin-resistant *S. aureus* (MRSA) strains. Butremycin was found to show very weak antibacterial activity against almost all strains while rifampicin was distinctly more active and Table 3 shows the MIC of both compounds.

**Table 3:** Antibacterial activity of butremycin against *Staphylococcus aureus* ATCC25923, *Escherichia coli* ATCC 25922 and a selection of methicillin-resistant *Staphylococcus aureus* (MRSA) strains.

Strain	Source of Isolate	MIC (µg mL^−1^)	
		Butremycin	Rifampicin
*S. aureus* ATCC 25923 ^a^	ATCC	50	0.003
*E. coli* ATCC 25922 ^a^	ATCC	50	0.01
SMRSA 105	Toe wound	>50	0.007
SMRSA 124	Open wound	>50	0.03
SMRSA 116	Knee abscess	>50	0.01–0.03
EMRSA 15	Urine infection	50	0.001–0.003

^a^ Laboratory strains; All clinical isolates were obtained from the NHS Grampian Microbiology Diagnostic Laboratory, Aberdeen Royal infirmary; Abbreviations: EMSRA = epidemic MRSA, SMRA = Scottish MRSA.

## 3. Experimental Section

### 3.1. General Experimental Procedures

NMR data were acquired on a Varian VNMRS 600 MHz spectrometer. High resolution mass spectrometric data were obtained using a Thermo Instruments MS system (LTQ XL/LTQ Orbitrap Discovery, Thermo Scientific, Bremen, Germany) coupled to a Thermo Instruments HPLC system (Accela PDA detector, Accela PDA autosampler and Accela pump). The following conditions were used: capillary voltage 45 V, capillary temperature 260 °C, auxillary gas flow rate 10–20 arbitrary units, sheath gas flow rate 40–50 arbitrary units, spray voltage 4.5 kV, mass range 100–2000 amu (maximum resolution 30,000). HPLC separations were carried out using a Phenomenex Luna reverse-phase (C18 250 × 10 mm, L × i.d.) column connected to a Waters 1525 Binary HPLC pump Chromatograph with a 2998 PDA detector, column heater and in-line degasser. Detection was achieved on-line through a scan of wavelengths from 200 to 400 nm. Diaion HP-20 was obtained from Resindion S.R.L., a subsidiary of Mitsubishi Chemical Co., Binasco, Italy. All solvents used throughout were HPLC-grade and purchased from Sigma-Aldrich (Munich, Germany) through a Ghana based agent (Huge Limited, Accra, Ghana). Sephadex LH-20 (25–100 μm) was purchased from GE Healthcare (Little Chalfont, UK). TLC Silica gel plates (60F_254nm_) were purchased from Merck KGaA (Darmstadt, Germany).

### 3.2. Identification of *Micromonospora* sp. K310

The Bacterial Genomic DNA Isolation Kit (Norgen Biotek, Ontario, Canada) was used for genomic DNA extraction from strain K310 according to the manufacturer’s protocol. PCR-mediated amplification of the 16S rDNA gene and purification of the PCR product were carried out following Chun and Goodfellow (1995) [16]. The almost complete (1455 bp) 16S rRNA gene sequence of strain K310 was determined using an ABI PRISM 3730 XL automatic sequencer. The identification of phylogenetic neighbors and calculation of pair-wise 16S rRNA gene sequence similarity were achieved using the EzTaxon-e server [17]. Multiple alignments with sequences from closely related species were performed by using the program CLUSTAL W in the MEGA5 software package [18]. Phylogenetic tree was constructed with the neighbor joining algorithm in MEGA5 [10,18]. Evolutionary distances were calculated using model of Jukes and Cantor (1969) [19]. Topologies of the resultant trees were evaluated by bootstrap analysis [20] based on 1000 re-samplings.

### 3.3. Isolation of *Micromonospora* sp. K310 and Preliminary Screening of the Secondary Metabolites

Sediment sample was collected from the Butre River in the Western Region of Ghana (coordinates: 4°49′43.73″N and 1°54′50.84″W). *Micromonospora* sp. K310 was isolated from the swampy mangrove sediment using the procedure outlined as follows. A small portion of the sediment was transferred into sterile 50 mL tubes, covered and the cap wrapped tightly with cellophane to prevent the entry of water or steam. The tube containing sample was then immersed in a water bath set at temperature 55 °C and heated for 3 h to eliminate non-sporulating bacteria. The sample was then suspended in 10 mL of sterile water under a clean bench and filtered using a previously autoclaved filter paper. The resultant filtrate was serially diluted to 10^−1^, 10^−2^ and 10^−3^ of the original volume. Subsequently, 50 μL of each of the three dilutions were transferred to previously prepared casein starch agar plates (agar, 15 g; soluble starch, 10 g; dibasic potassium phosphate, 2 g; potassium nitrate, 2 g; sodium chloride, 2 g; casein, 0.3 g; magnesium sulfate heptahydrate, 0.05 g; calcium carbonate, 0.02 g; iron sulfate heptahydrate, 0.01 g; sea salt, 33 g and 1 L of tap water) supplemented with nalidixic acid and nystatin at 2.5 μg/L each. Different colonies of *Micromonospora* sp. were observed after three weeks of incubation at 28 °C. Subsequently, each colony was repeatedly transferred and streaked on different fresh casein starch plates with the aid of autoclaved tooth picks until pure strains were obtained (Supplementary Figure S13). One individual bacteria colony from strain K310 was fermented in 50 mL of starch casein liquid media using previously autoclaved 250 mL Erlenmeyer flasks plugged with non-absorbent cotton wool. The culture was allowed to grow for ten days at 28 °C with continues agitation at 200 rpm. Diaion HP-20 (50 g/L) was added to the culture three days prior to harvesting. The culture was then filtered under suction through a piece of glass wool placed in a Buchner funnel and the filtrate was discarded. The Diaion HP-20 resins were then soaked repeatedly and alternatively in CH_3_OH and CH_2_Cl_2_ and the resultant extracts were combined and dried under vacuum to give the crude extract. The extract was then subjected to HPLC/HRESIMS analysis. Analysis of the resultant data from HPLC/HRESIMS revealed two very well resolved peaks with [M + H]^+^ and [M + Na]^+^ ions seen for the two compounds at *m*/*z* 299.0810 and 495.2781 respectively (Supplementary Figure S14). These masses were entered as independent queries in the Natural Products Identifier AntiBase 2013 software to check whether these molecules were new or novel. The search came with no relevant hits and the *Micromonospora* sp. K310 was tagged as one of the interesting strains for further investigation.

### 3.4. Large Scale Fermentation of K310

A pure colony of *Micromonospora* sp. K310 was used to inoculate a 250 mL Erlenmeyer flask containing 50 mL of liquid starch casein media as described above but without agar. After seven days of incubation at 28 °C with continuous agitation at 200 rpm, the culture was used to inoculate two 3 L conical flasks containing already autoclaved 1 L starch casein liquid media and plugged with non-absorbent cotton wool. The two 3 L flasks were incubated at 28 °C with continuous agitation at 250 rpm for three weeks. Diaion HP-20 resin (50 g/L) was added under sterile conditions using a serological pipette to both flasks after three weeks and returned back to the incubator set at 28 °C for another one week of continuous agitation at 250 rpm. After four weeks of incubation, the two 1 L cultures were harvested and filtered under pressure using a piece of glass wool placed in a Buchner funnel. The filtrate was discarded and the residue consisting mainly of the Diaion HP-20 resin with adsorbed organics was repeatedly and alternatively extracted with CH_3_OH and CH_2_Cl_2_. The CH_3_OH and CH_2_Cl_2_ extracts were combined and concentrated under reduced pressure to give 960 mg of a light yellow total crude extract (TCE).

### 3.5. Extraction, Isolation and Purification of Compounds

The TCE was suspended in 150 mL of H_2_O and extracted three times with the same volume of CH_2_Cl_2_. The CH_2_Cl_2_ extract was concentrated under reduced pressure and transferred into a vial for subsequent analysis and separation while the water layer was extracted with the same volume of 2-butanol once. The 2-butanol layer was also concentrated under reduced pressure to give a water-butanol fraction WB (456 mg). This extract was subsequently loaded on a gravity column packed with Sephadex LH-20 and eluted with a 50/50 mixture of CH_3_CN and CH_3_OH. Seven fractions were collected from the Sephadex LH-20 run and labeled SF1-7. HPLC/HRESIMS showed that SF3 (120 mg) and SF5 (96 mg) contained the compounds of interest. These fractions were therefore subjected to HPLC separation and purification using a Phenomenex Luna C_18_ column (C18 250 × 10 mm, L × i.d.). Gradients of H_2_O:CH_3_CN (100% H_2_O to 100% CH_3_CN in 30 min and hold for 20 min) were used as eluent with column flow rates set at 1.5 mL/min to afford butremycin (46.6 mg) and compound **3** (57.0 mg). 

Compound **2** (butremycin): Pale yellow crystals; UV (CH_3_OH/0.1%HCOOH) 227, 325 nm; IR (neat) ν_max_ 3368, 2956, 2924, 2853, 1602, 1479, 1417, 1093, 1029 and 694 cm^−1^; For ^1^H, ^13^C NMR data, see Table 1; HRESIMS (positive mode) *m*/*z* 495.2781 [M + H]^+^; calcd. for C_29_H_39_N_2_O_5_.

Compound **3**: Deep yellow crystals; UV (CH_3_OH/0.1%HCOOH) 248 nm; IR (neat) ν_max_ 3392, 2926, 1698, 1589, 1548, 1418, 1377, 1344, 1219, 1127, 1093, 1050 cm^−1^; For ^1^H, ^13^C NMR data, see Table 2; HRESIMS (positive mode) *m*/*z* 299.0810 [M]^+^; calcd. for C_11_H_15_N_4_O_4_S^+^.

### 3.6. Antibacterial Activity

The antibacterial activity of butremycin was evaluated against *S. aureus* ATCC 25923, *E. coli* ATCC 25922 and a panel of methicillin-resistant *S. aureus* clinical isolates obtained from the NHS Grampian Microbiology Diagnostic Laboratory using slight modifications of the previously described method [21]. In brief, bacterial strains were grown in Müller-Hinton (MH) broth [22] to early stationary phase and then diluted to an OD_620_ = 0.1. The assays were performed in a 96-well micro titer plate format. Rifampicin and butremycin were dissolved in DMSO (Sigma, Munich, Germany), and the effect of different dilutions in broth on the growth was assessed after 18 h incubation at 37 °C using a Labsystems iEMS Reader MF plate reader at OD_620_. The MIC was determined as the lowest concentration showing no growth compared to the MH broth control. DMSO up to 10% was shown to have no antibacterial effect.

## 4. Conclusions

Butremycin, the (3-hydroxyl) derivative of ikarugamycin was isolated from *Micromonospora* sp. K310 isolated from a sediment collected from the Butre River which is situated in the Western Region mangroves of Ghana. This represents the first report of a PTM from a *Micromonospora* sp. The compound, butremycin was found to exhibit weak antibacterial activity against the Gram-positive *S. aureus* ATCC 25923, the Gram-negative *E. coli* ATCC 25922 and a panel of clinical isolates of methicillin-resistant *S. aureus* strains. In addition to this, the new species of *Micromonospora* was found interestingly to be actively involved in the synthesis of compound **3**. This represents the first report of the isolation of a 5′-methylthioinosine derivative from a *Micromonospora* sp., notwithstanding the fact that recently this compound has been implicated in a two step 5′-methylthioinosine degradative pathway in *Pseudomonas aeruginosa* that is required for quorum sensing.

## Supplementary Files

Supplementary File 1 Supplementary Information (PDF, 1373 KB)
