# Supplementary material for: Butremycin, the 3-Hydroxyl Derivative of Ikarugamycin and a Protonated Aromatic Tautomer of 5′-Methylthioinosine from a Ghanaian Micromonospora sp. K310
Source: Mar Drugs. 2014 Feb 14;12(2):999–1012. doi: 10.3390/md12020999 (PMC3944527; doi:10.3390/md12020999)

Supplementary Information

Figure S1. MS fragmentation pattern for butremycin (**2**) obtained from the HPLC/HRESIMS data of pure compound.
Figure S2. ^{1}H NMR spectrum of butremycin (**2**).
Figure S3. ^{13}C NMR spectrum of butremycin (**2**).
Figure S4. gHSQCAD spectrum of butremycin (**2**).
Figure S5. ^{1}H-^{1}H gCOSY spectrum of butremycin (**2**).
Figure S6. HMBCAD spectrum of butremycin (**2**).
Figure S7. TROESY spectrum of butremycin (**2**).
Figure S8. ^{1}H NMR spectrum of compound (**3**).
Figure S9. ^{13}C NMR spectrum of compound (**3**).
Figure S10. gHSQCAD spectrum of compound (**3**).
Figure S11. ^{1}H-^{1}H gCOSY spectrum of compound (**3**).
Figure S12. HMBCAD spectrum of compound (**3**).
Figure S13. *Micromonospora* sp. K310 growing on starch casein agar with sea salts.
Figure S14. PDA/UV profiles obtained from the HPLC/HRESIMS data of the crude extracts of *Micromonospora* sp. K310.

Figure S1. MS fragmentation pattern for butremycin (**2**) obtained from the HPLC/HRESIMS data of pure compound.

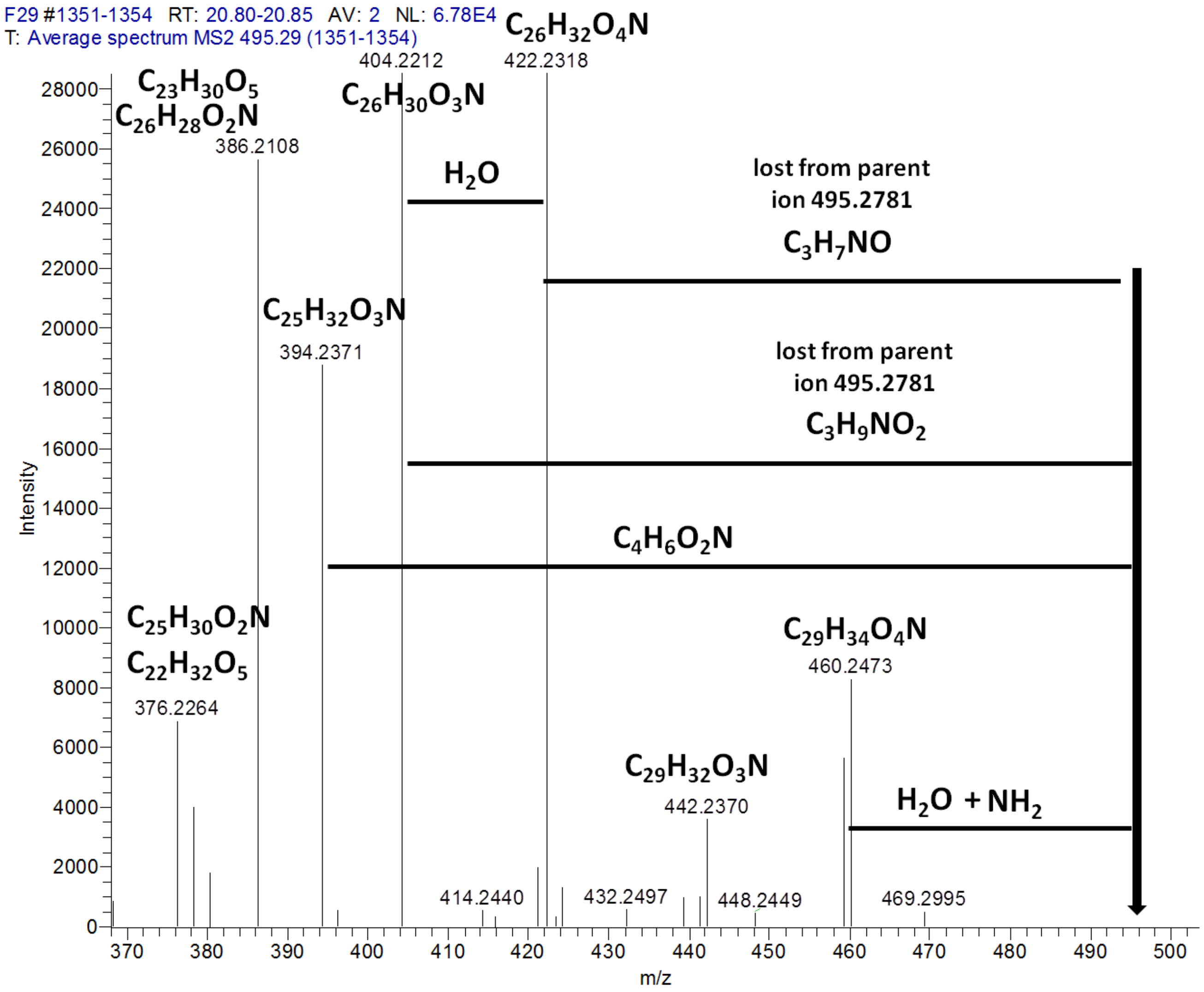

Figure S2. ^{1}H NMR spectrum of butremycin (**2**).

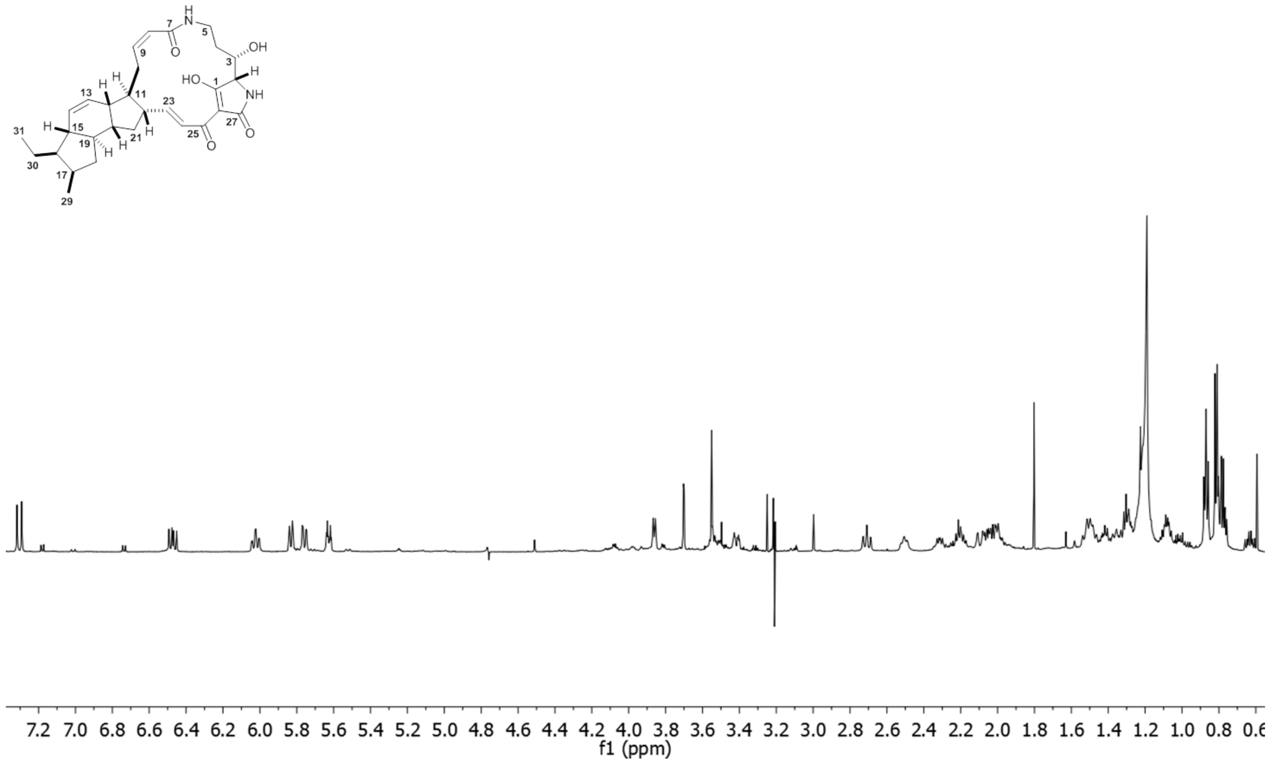

Figure S3. ^{13}C NMR spectrum of butremycin (**2**).

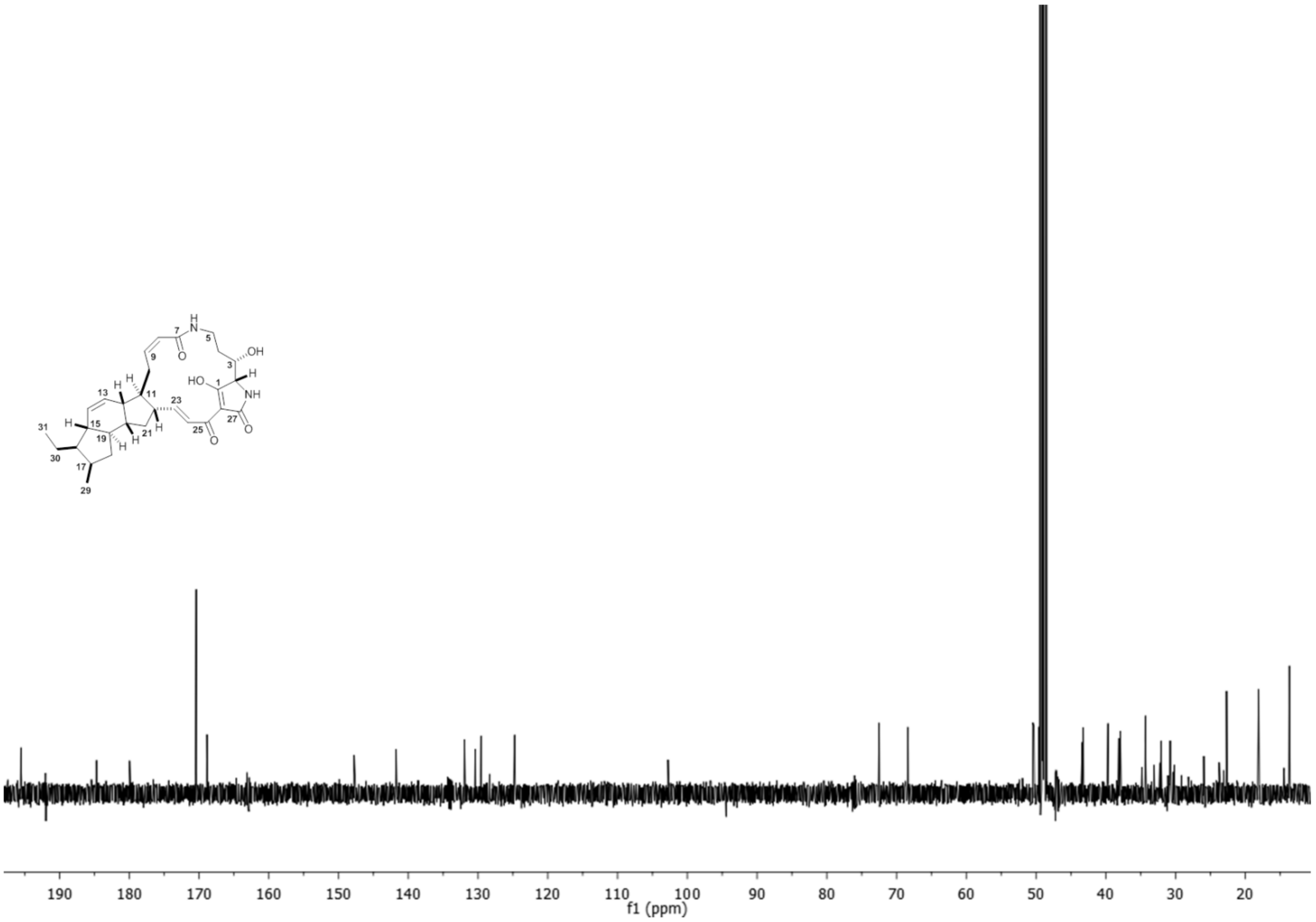

Figure S4. gHSQCAD spectrum of butremycin (**2**).

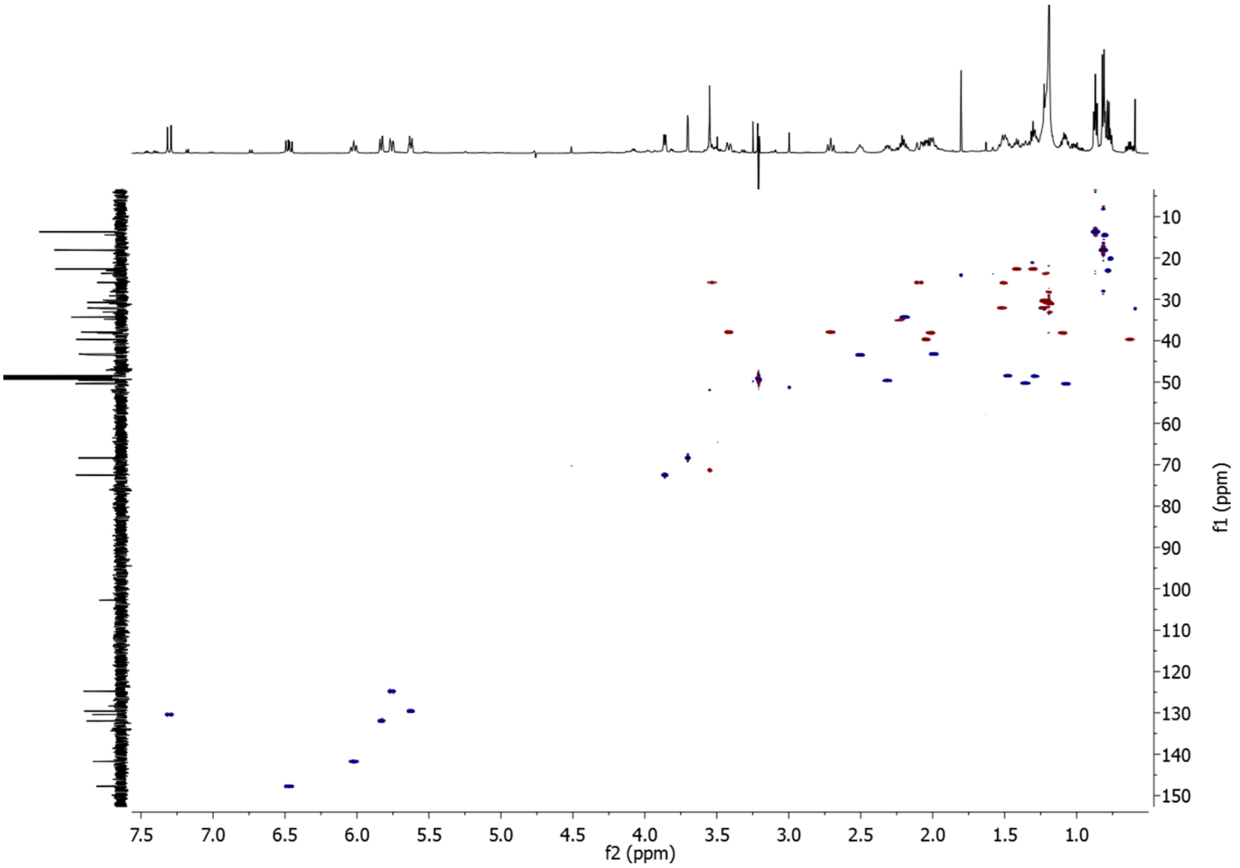

Figure S5. ¹H-¹H gCOSY spectrum of butremycin (**2**).

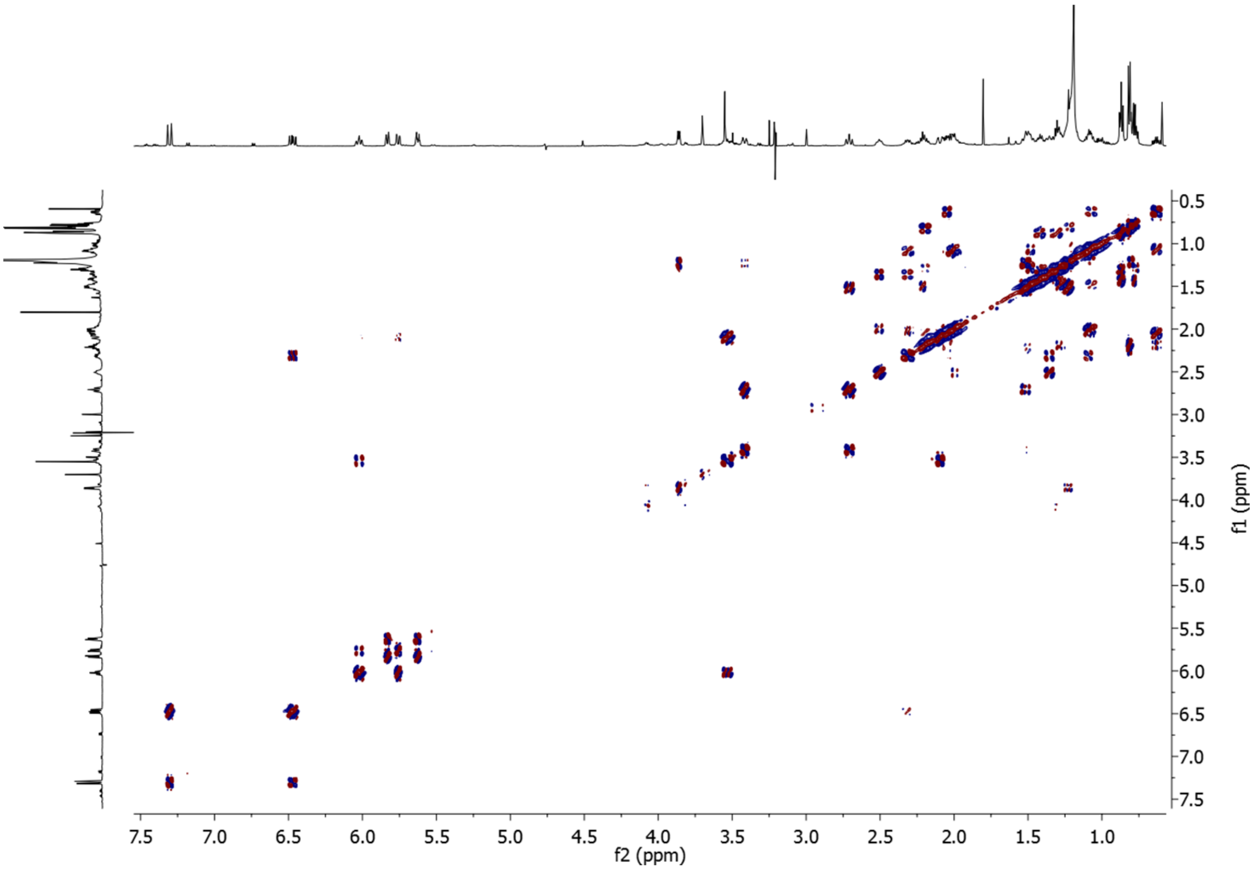

Figure S6. HMBCAD spectrum of butremycin (**2**).

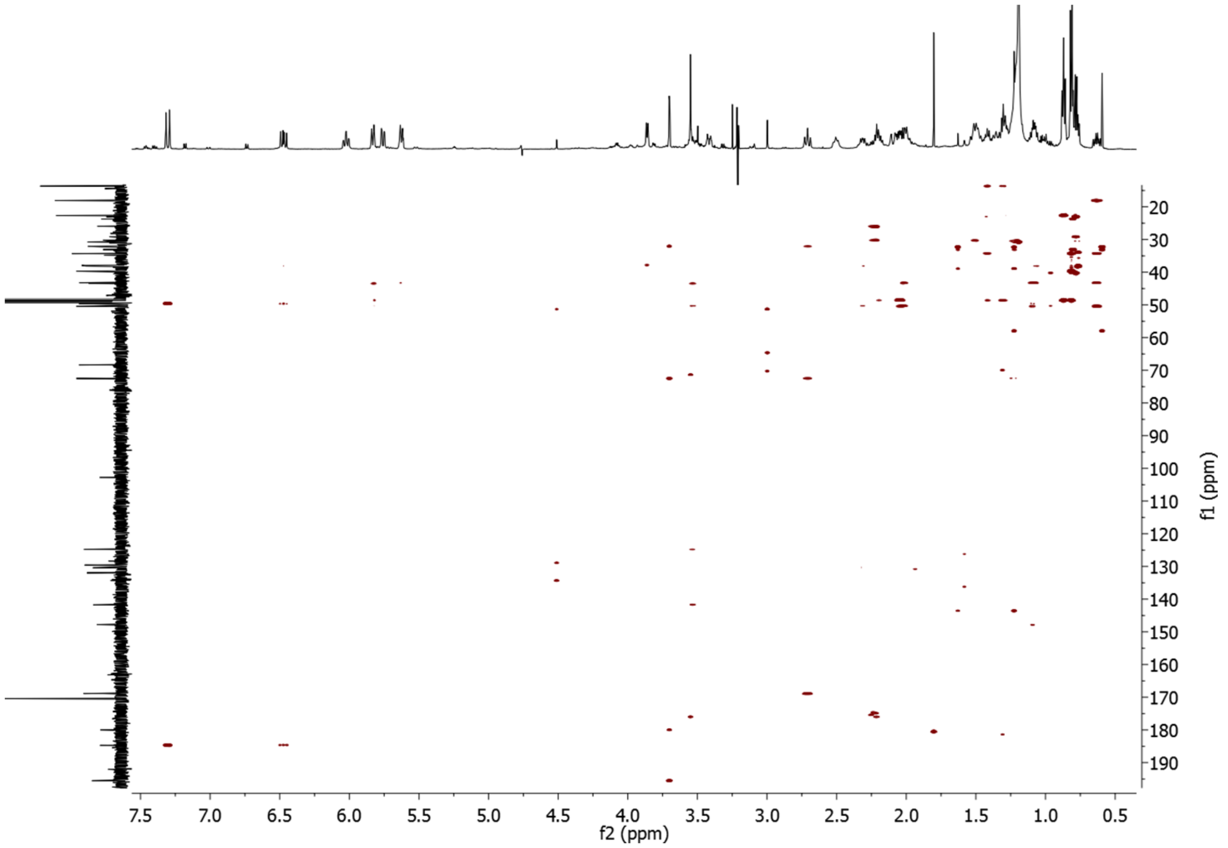

Figure S7. TROESY spectrum of butremycin (**2**).

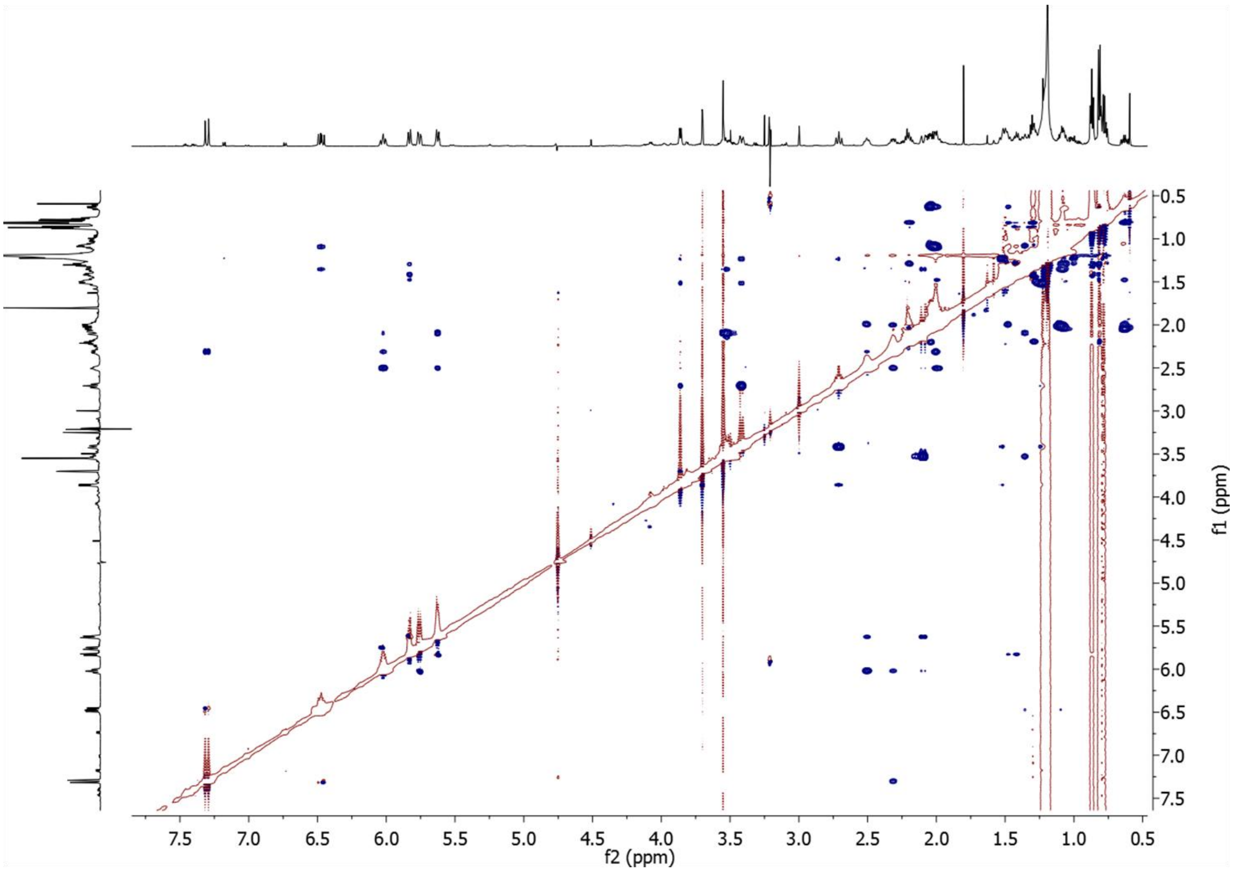

Figure S8. ^{1}H NMR spectrum of compound (**3**).

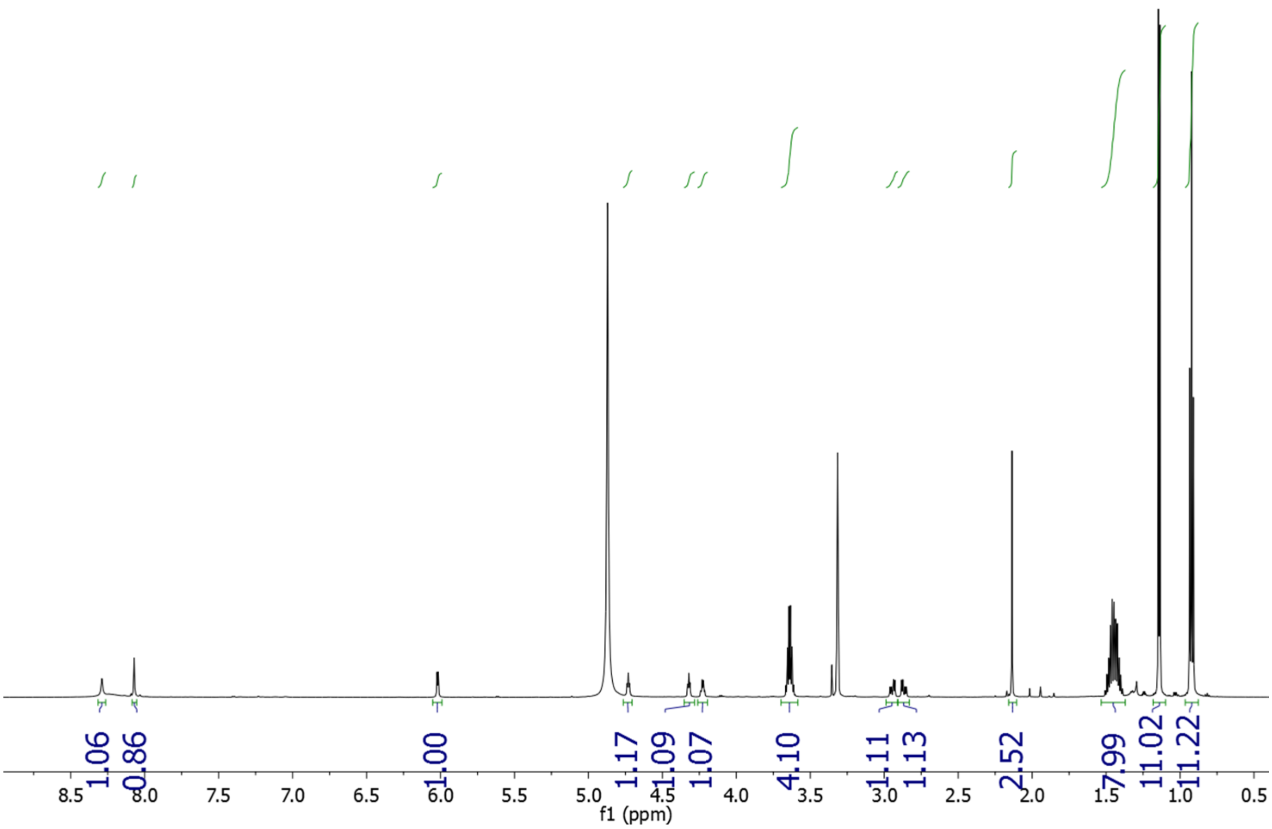

Figure S9. ^{13}C NMR spectrum of compound (**3**).

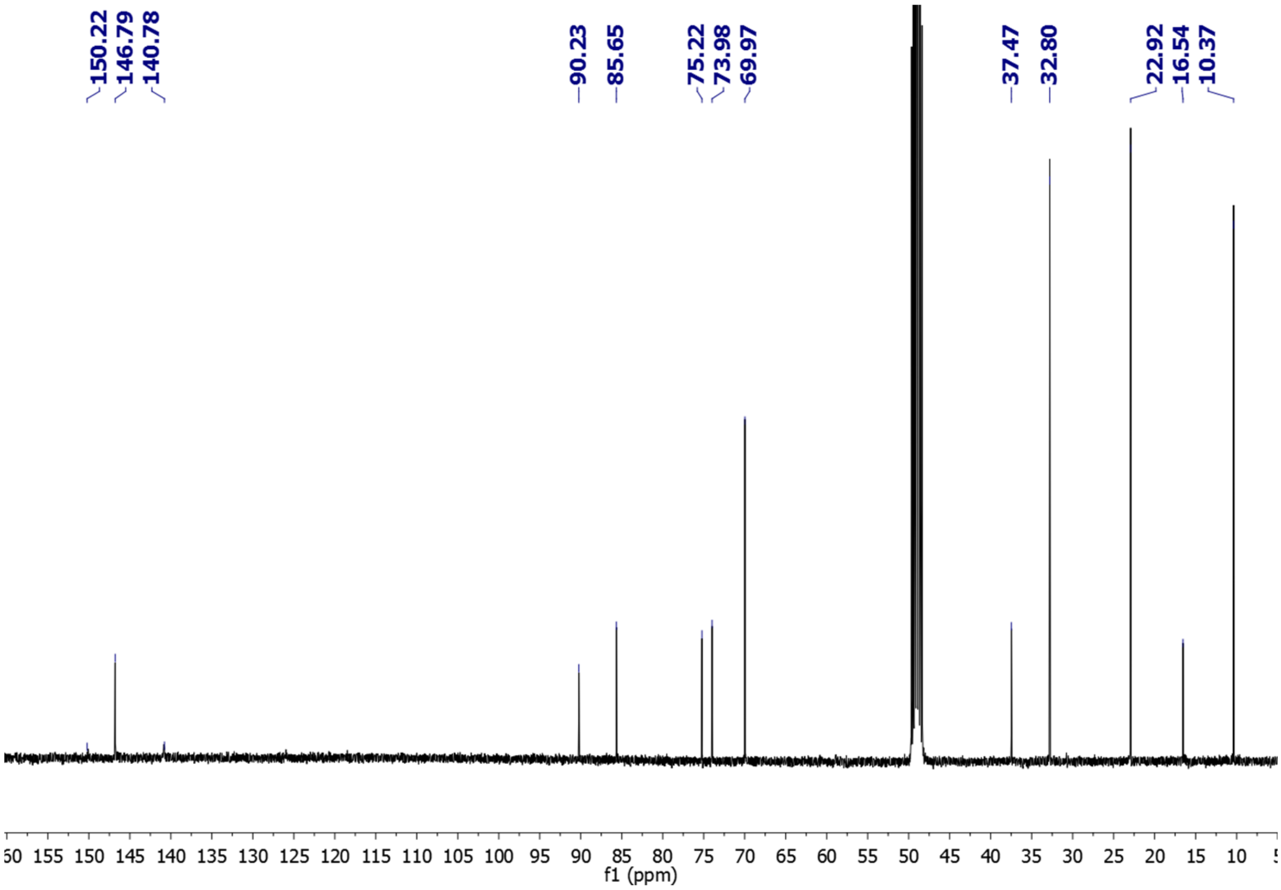

Figure S10. gHSQCAD spectrum of compound (**3**).

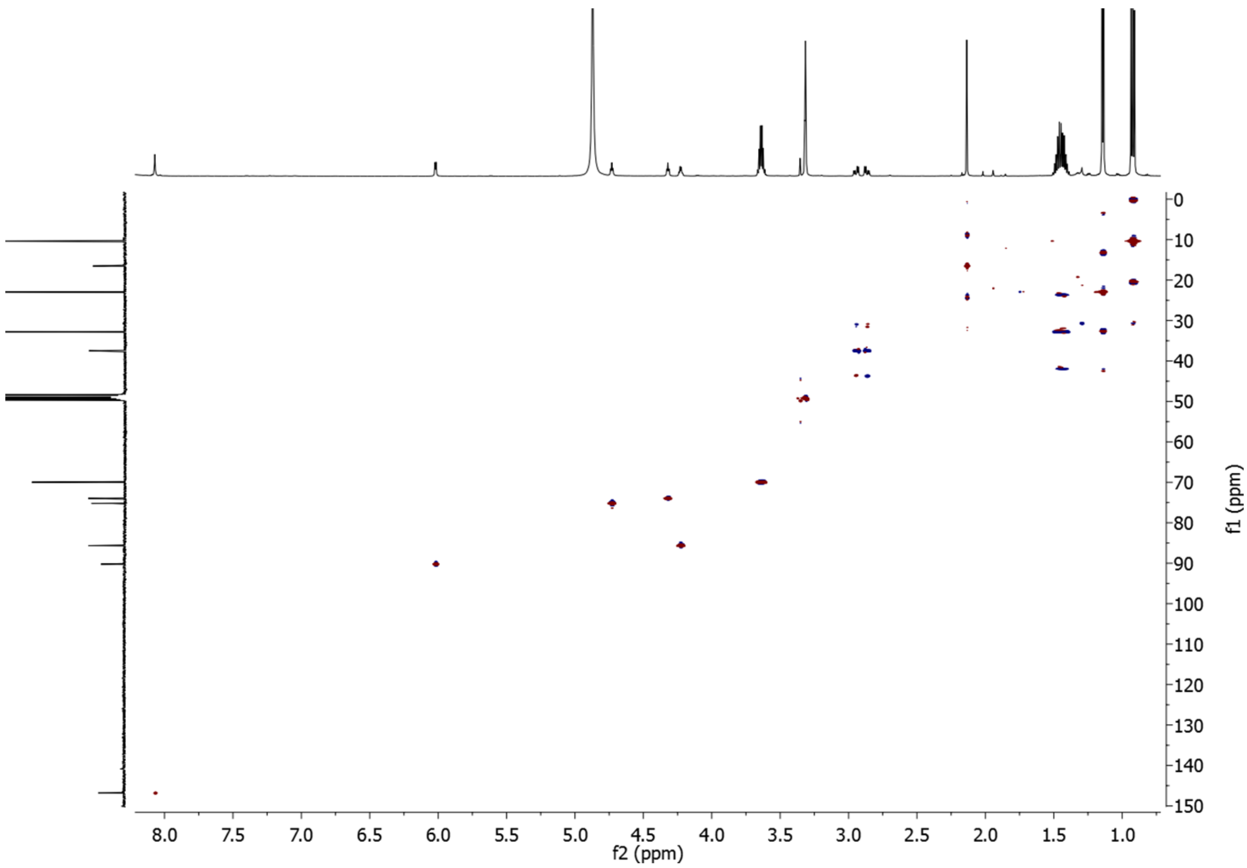

Figure S11. ^{1}H-^{1}H gCOSY spectrum of compound (**3**).

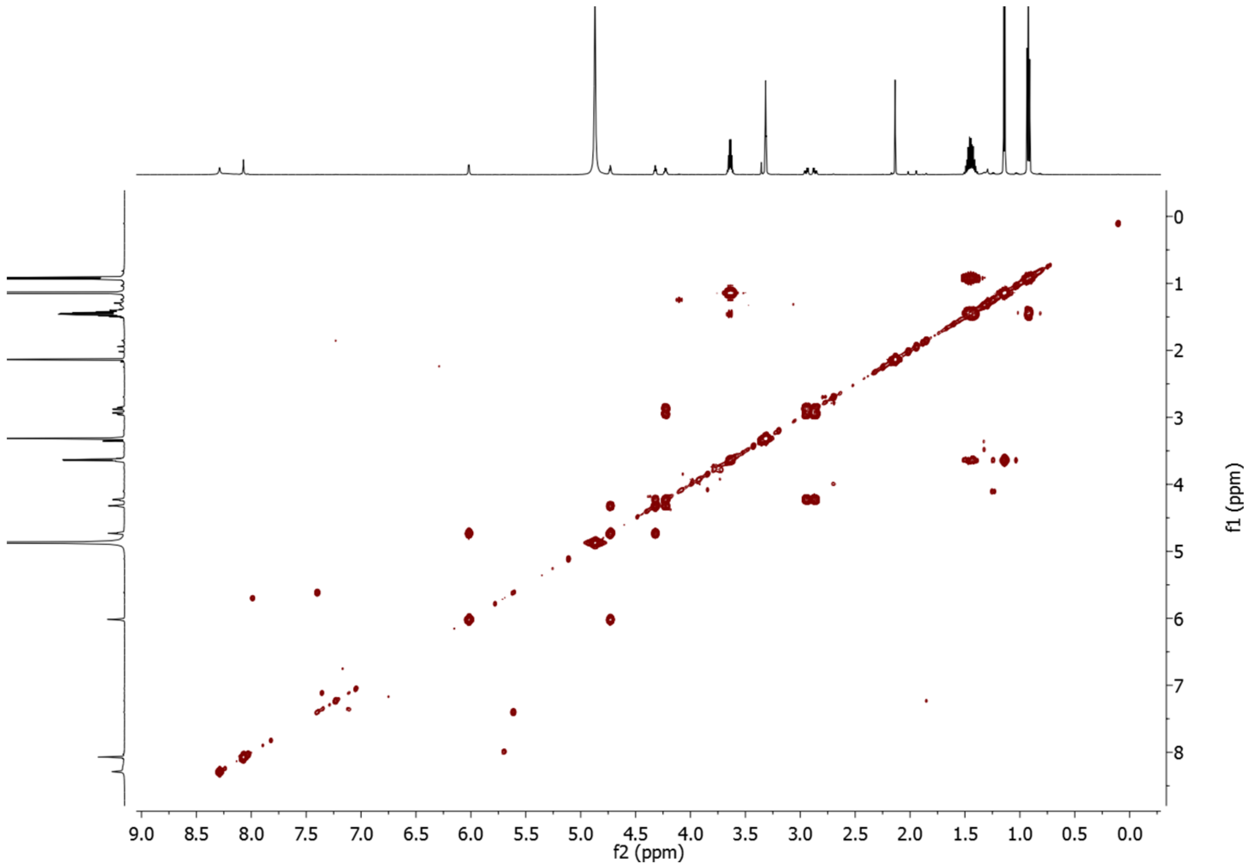

Figure S12. HMBCAD spectrum of compound (**3**).

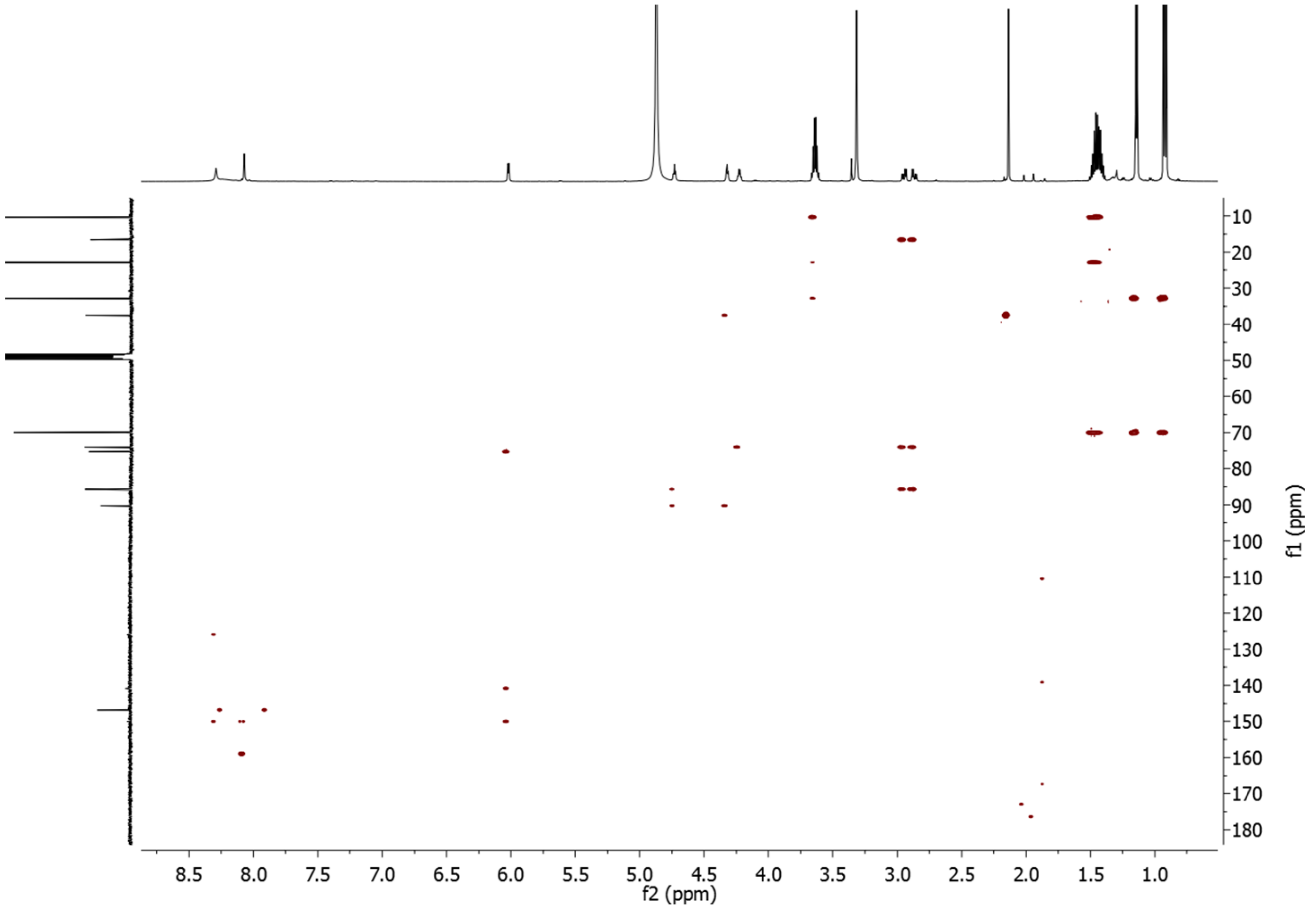

Figure S13. *Micromonospora* sp. K310 growing on starch casein agar with sea salts.

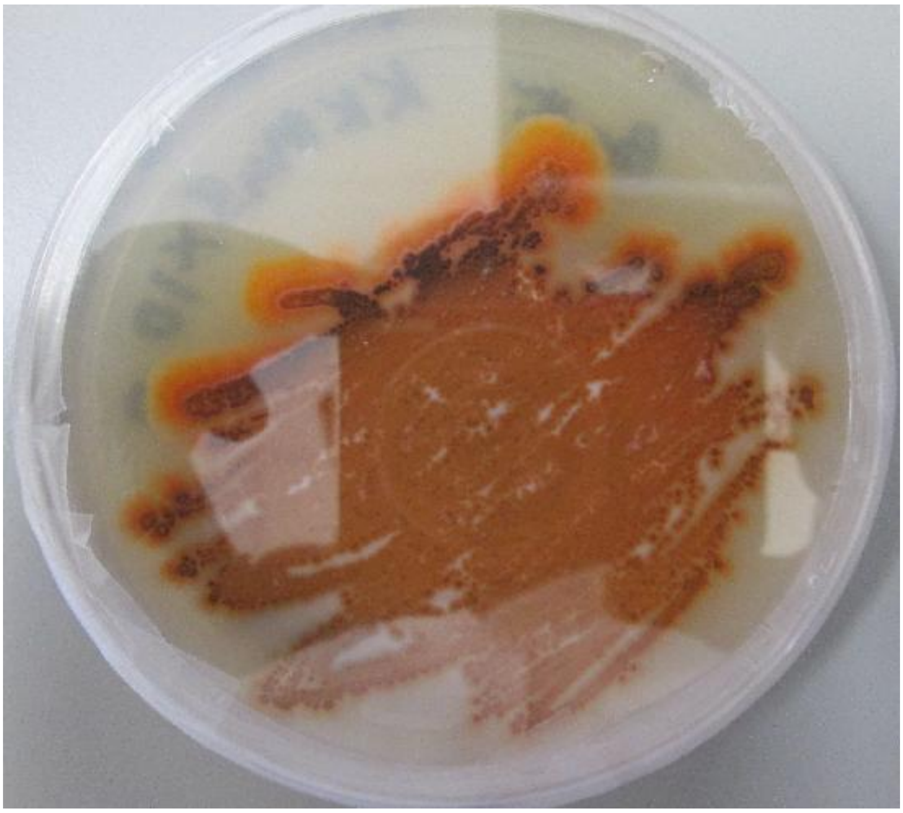

Figure S14. PDA/UV profiles obtained from the HPLC/HRESIMS data of the crude extracts of *Micromonospora* sp. K310.

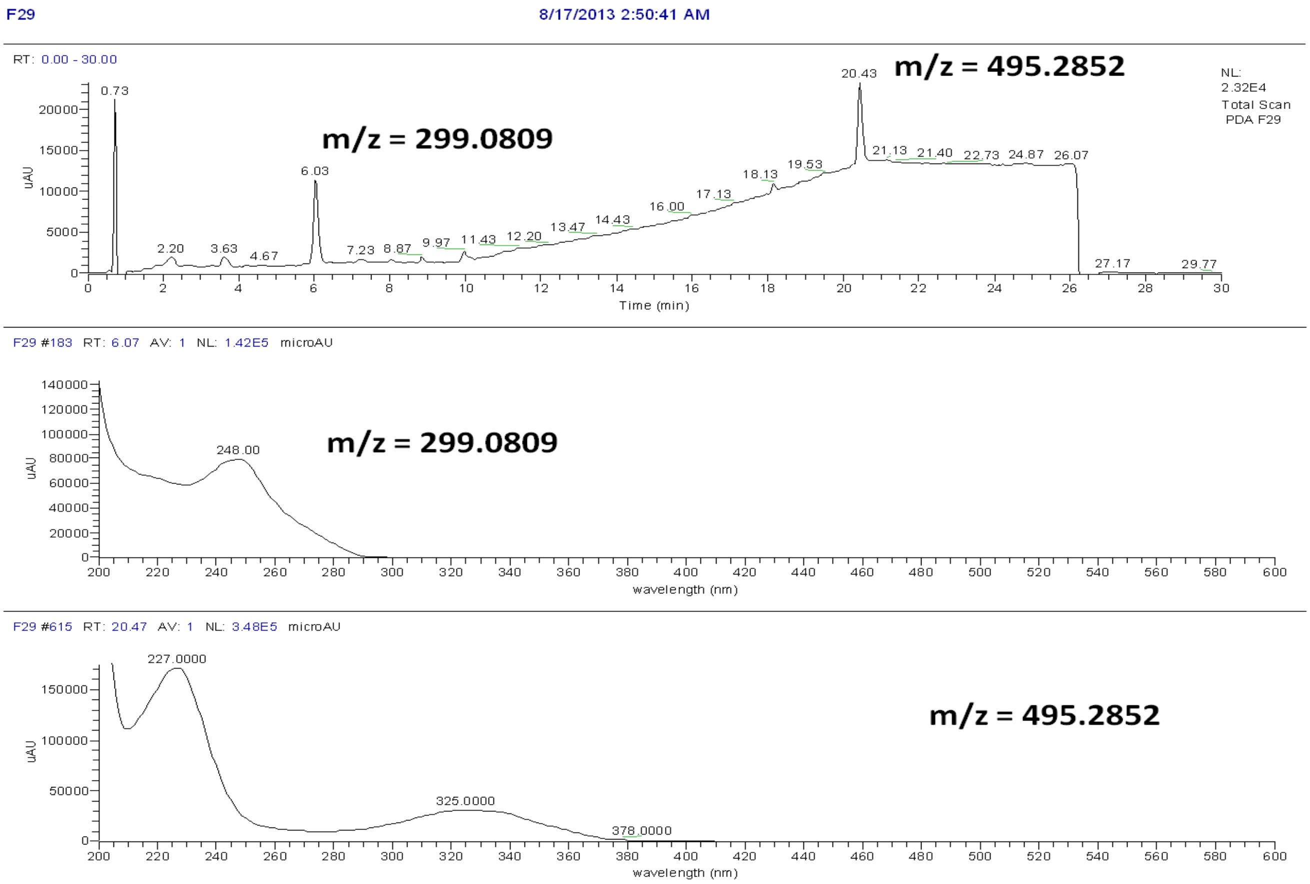

Supplement: Supplementary File 1 — Supplementary Information (PDF, 1373 KB) [file marinedrugs-12-00999-s001.pdf]
